# Cognitive Plasticity in Young-Old Adults and Old-Old Adults and Its Relationship with Successful Aging

**DOI:** 10.3390/geriatrics3040076

**Published:** 2018-10-29

**Authors:** Elena Navarro, M. Dolores Calero

**Affiliations:** 1 Department of Personality, Psychological Assessment and Treatment, Faculty of Psychology, University of Granada, 18071 Granada, Spain; 2 CIMCYC, Research Center on Mind, Brain and Behavior, University of Granada, 18071 Granada, Spain; mcalero@ugr.es

**Keywords:** cognitive plasticity, successful aging, longevity, Auditory Verbal Learning Test—Learning Potential (AVLT-LP), young-old adults, old-old adults

## Abstract

The general objective of this study was to analyze cognitive plasticity as a variable related to successful aging in a group of young-old adults and old-old adults using the Auditory Verbal Learning Test—Learning Potential (AVLT-LP). Method: A total of 569 persons, with mean age 76.67 years (379 between the ages of 65 and 80 years, and 190 older than age 80). They were assessed with a socio-health questionnaire, with the AVLT-LP, and with the Spanish version of the Mini Mental State Examination. Results: The results showed significant differences on the test, in favor of the younger group, while the over 80 group gave poorer performance and showed less cognitive plasticity. With relation to gender, slight differences appeared in favor of the women, on the first four test trials, but not on the last two, nor in delayed recall or cognitive plasticity. As for cognitive status, the results showed significantly better task performance levels in healthy elders, as well as greater plasticity. Nonetheless, certain persons with high plasticity were also found among those with cognitive impairment. Conclusions: The data obtained here offers evidence for the importance of cognitive plasticity in elders and its relation to longevity and successful aging. It also provides information about the influence of variables like age, gender and cognitive status on a verbal memory and plasticity assessment task that is in wide use today.

## 1. Introduction

The increasing elderly population in Western countries is an unprecedented fact in our recent history. For example, it is expected that by the year 2020, the number of persons over age 65 in Europe and the United States will comprise 20% of the population [1,2].

Aging is usually accompanied by losses in both cognitive and physical abilities [3]. In relation to cognitive loss, impairment is usually observed in skills related to fluid intelligence, such as working memory and episodic memory, reasoning and spatial orientation [4,5,6]. Despite the above, the literature indicates that there are important inter-individual differences in cognitive functioning during old age [7,8]. Following this line, many studies have demonstrated potential for learning and cognitive improvement by exploring the concept of cognitive plasticity [9,10,11]. 

The concept of cognitive plasticity refers to intraindividual modifiability, and is defined by the authors [12,13] as intellectual performance in old age under optimized conditions that do not normally exist in the person’s daily life or in the standard assessment situations of classical intelligence tests [13]. The term cognitive plasticity takes on particular importance in gerontological literature, where one is able to determine to what extent a person can improve their performance after a training phase [14]. According to Lövden et al. [5], it is an adaptive response to cognitive demands that exceed one’s cognitive resources and reflects the individual’s potential for improvement after training [14]. When we speak of cognitive plasticity, its relationship to the concept of brain plasticity must be mentioned [15,16]. According to Fernández-Ballesteros et al. [17], the two terms refer to different levels of the same construct, such that we find brain plasticity at the neurobiological level, and cognitive plasticity at the behavioral level. Cognitive plasticity is observable with assessment techniques that involve a training phase, and it has physiological correlates to brain plasticity [18,19]. For cognitive plasticity assessment, one of the most commonly used procedures is the testing-the-limits approach, also called dynamic or learning potential assessment [20,21], an alternative assessment procedure to traditional or static assessment of cognitive functioning [16]. Dynamic assessment is important in that it enables us to distinguish a person’s performance level under standard conditions (as measured by traditional or static assessment) from their performance capacity under optimized conditions [12]. More specifically, this methodology consists of: (1) Presenting a cognitive task in a standard situation (pretest phase), thereby establishing the person’s baseline level of functioning; (2) offering a training phase on the type of task being assessed; and finally, (3) a new assessment under the standard conditions (posttest phase) [22]. Any improvement after the training phase, that is, the difference between the posttest and the pretest (gain score), is used as an indicator of cognitive plasticity, because it indicates to what extent the person has benefitted from the training [17]. Concerning the differences between the two assessment procedures, we refer the reader to the meta-analysis by Swanson and Lussier [23], where they compared the effects of traditional assessment to dynamic assessment, and found that the training phase included in dynamic assessment procedures significantly improved performance when compared to simply repeating the test, or practicing the task [23].

In the case of an older population, it is advisable that tests not be very long, in order to avoid fatigue and the resulting loss of attention. For this reason, adapted assessment tasks are often used to assess and train memory skills or skills that are close to fluid intelligence, uncontaminated by cultural aspects [24].

Using this assessment methodology, previous studies have demonstrated the presence of plasticity in healthy older adults, the absence of plasticity in older adults with cognitive impairment [25], the implications of plasticity with respect to the cognitive evolution of older adults [26], and its effectiveness as an indicator of impairment [3]. Age-associated differences in plasticity were also found [27,28]. Thus, according to several authors, while there may be continued plasticity in a group of old-old adults, it is less present than in a group of young-old adults [29,30]. To differentiate between the group of young-old adults and old-old adults, Baltes’s team [31] initially proposed a cutoff of 80–85 years, given that this is the age when 50% of persons in this generational cohort would have died. This classification of young-old adults (under 80 years) and old-old adults (over 80 years) is often used in specialized gerontological literature and is necessary, due to the extension of this phase of life, where there are important inter-individual differences that require different phases to be distinguished [31,32,33,34]. Multiple investigations justify this classification based on studies where the young-old adults usually show good levels of cognitive and physical functioning [35,36], while the group of old-old adults often present generalized cognitive impairment [31,37,38,39].

Based on the literature review, we chose the Auditory Verbal Learning Test-Learning Potential (AVLT-LP) [40], adapted for an elderly Spanish population [41], as our cognitive plasticity assessment measure for the present research study. In its traditional version, the AVLT [42] assesses episodic memory, a cognitive function whose decline seems to indicate cognitive impairment [6,8,38]. In its dynamic version (learning potential version), the main objective of the AVLT-LP is to assess a person’s cognitive plasticity. Several studies have demonstrated that it is a valid tool to evaluate plasticity in older persons and in other populations [40,43]. Prior research has shown its concurrent validity with other measures of cognitive plasticity [41], as well as its predictive validity for cognitive evolution in elders with and without cognitive impairment [26,44]. The training administered in the intermediate phase of this test is a mediational procedure that involves positive reinforcement regarding performance, offering information about right and wrong responses, and the reasons why, motivation for improvement, and making the person aware of the most adequate strategies for solving the task. This feedback regarding performance, followed by the self-guided retest [29] seems to be one of the most adequate strategies for determining plasticity in elders, as well as for producing greater maintenance of effects over time, and greater generalization of effects [29].

The general objective of this investigation was to analyze cognitive plasticity in a large sample of elders from the south of Spain, using the AVLT-LP test. Specific objectives were: (1) To analyze the influences of age, gender and cognitive status on AVLT-LP performance and on cognitive plasticity in the test; and (2) analyze the presence or absence of plasticity according to age range (young-old adults and old-old adults), gender and cognitive status.

## 2. Materials and Methods

### 2.1. Participants

There were 569 participants, of whom 219 (38.5%) were men and 350 (61.5%) were women. Average age was 76.67 ± 8.11, age range was 60–98 years. Regarding educational level, 349 (61.34%) did not have formal studies, 125 (21.96%) had attended primary school; and 95 (16.7%) had a secondary education or higher. The participants came from senior residences in two provinces of southeast Spain. Participants were divided according to the following variables: (1) Age range, (2) cognitive status, (3) gender, and (4) cognitive plasticity status as described in the ‘Procedures’ section.

### 2.2. Instruments

*Socio-health questionnaire:* Designed for the present and previous studies [41,44] with the objective of obtaining a variety of socio-demographic and health-related data.

*Auditory Verbal Learning Test of Learning Potential* [40]: As indicated in the introduction, this test is an adaptation of the classical AVLT verbal memory task, by Rey [42]. In its dynamic version (learning potential version), the AVLT-LP assesses cognitive plasticity. A list of 15 words is presented six times consecutively. The initial two presentations (A1, A2) are considered the pretest and follow the standard procedure; the following two presentations (A3, A4) constitute the training, where reinforcement and performance feedback are offered, repetition of the words not recalled, and verbalizations to help focus the participant’s attention on the task. The two final presentations of the list of words (A5, A6) represent the posttest and follow the standard procedure. Once the test was completed, and after doing certain noncognitive activities (for example, informal chatting) that served as interference, the participant was again asked to repeat the words from the list without any assistance (A7, delayed recall). In this investigation, we made use of the following measurements obtained from this test: (1) Gain score, that is, the difference between the post-test and pre-test scores (AVLT-LP gain score), (2) the delayed recall score (A7: AVLT-LP delayed recall), (3) the performance obtained on each test trial (A1, A2, A3, etc.) and (4) the difference between the delayed recall trial and the first trial (A7 − A1). The AVLT-LP test has been validated by several authors in a Spanish population of elders, similar to the participants of the present study [20,41,45], and the AVLT-LP gain score has become established as a measure of cognitive plasticity in different populations, such as people with schizophrenia or dementia [43].

*Mini-examen-cognoscitivo* (*MEC*) [mini cognitive examination] [46], a Spanish version of the Mini-Mental State Examination (MMSE) [47]. This version of the test is an adaptation of the original task, developed in Spain by Lobo et al. [46], and assesses the same cognitive functions as the original test (temporo-spatial orientation, immediate and long-term recall, attention, calculation, language, abstract reasoning and praxis) and adds an additional attention task and another reasoning task. These two additional tasks bring the final score up to a possible maximum of 35 points. Prior studies have shown diagnostic agreement between the two tests [48], while the MEC is more sensitive to detecting cognitive status in an older Spanish population with a low level of education, as in the study presented here [48,49,50]. The final score obtained from the test is normally used as a global index and as a follow-up method for measuring the evolution of cognitive functions in processes, such as cognitive impairment and dementia. We used standards developed for the Spanish population [49] to establish criteria for the presence or absence of cognitive impairment. We confirmed the validity of these standards in a previous study [50].

### 2.3. Procedures

The investigation was carried out at different retirement homes in the south of Spain. Retirement homes in Spain are institutions that house older people on a temporary or permanent basis (in most cases due to some type of dependency). These centers offer gerontological services in psychological, social and health care. These services are overseen by a team of professionals trained in gerontology. The principal investigators of this study contacted management of the retirement homes in order to present the research, and, if they wished to collaborate, to request that they select residents that met the following characteristics: At least 60 years of age, absence of serious disease or dementia, and absence of motor or sensory deficits that would hinder them in carrying out the study tasks. The selected participants were informed about the characteristics of the study, and those who decided to participate gave their informed consent. Assessment was carried out in a session lasting approximately one hour, during which the above tests were administered. The assessment was carried out by specialized psychologists.

Keeping in mind the study objectives, the sample was divided according to two factors: (1) Age range and (2) cognitive status. (1) Two levels were established for age range, using a cutoff at age 80, following the criterion of prior research [28,31]: 60–80 years old (young-olds) (*n* = 379, *M* = 72.06 ± 5.27), and over 80 years old (old-olds) (*n* = 190, *M* = 85.88 ± 3.73, age range: 81–98). Two groups were established according to cognitive status: Old adults with cognitive impairment (*n* = 217, *M* = 20.24 ± 3.19) and healthy old adults (*n* = 352, *M* = 29.89 ± 2.96). For the cognitive status classification, the MEC score was taken as a reference with a cut-off score of 25 out of the maximum 35, following standardized criteria for a Spanish population with a similar educational level [49,50]. In a second phase, two groups were established according to: (1) Gender—men (*n* = 219) and women (*n* = 350); and (2) cognitive plasticity status. For the cognitive plasticity classification, we used the algorithm from Schöttke, Bartram and Wiedl [51], whereby a subject is classified as a gainer, or having high plasticity, when his or her improvement (pre–post difference in score, namely, AVLT gain score) is greater than 1.5 S.D. of the pretest score. According to this classification, two groups were established: Old adults with high plasticity (*n* = 242, AVLT gain score mean = 5.66 ± 1.4) and old adults with low plasticity (*n* = 327, AVLT gain score mean = 1.92 ± 1.15).

### 2.4. Statistical Analysis

First, we used the Kolmogorov-Smirnov and Levene statistical tests to check the assumptions of normality and homogeneity, respectively, of each of the dependent variables that were assessed. Once results were analyzed and the sample was seen to fulfill these assumptions, the following analyses were carried out: (1) To analyze between-group differences, a general linear univariate model was established. The factors (age range, gender and cognitive status) were applied to the model for all the variables (AVLT-LP trials and AVLT-LP gain score) considered to be dependent variables; (2) to analyze between-group differences between the young-old and the old-old adults with and without cognitive impairment, we used multivariate analysis of variance (MANOVA) with two factors for all the AVLT-LP measures that were dependent variables. In this case, the statistic chosen for these analyses was Wilks’ Lambda, because the groups were assumed to differ in more than one variable, and we applied a Bonferroni correction to control the overall Type I error rate. In order to control for the effect of educational level, this variable was included as a covariate in the analyses; (3) the chi-square test was used to analyze sample distribution according to participants’ cognitive plasticity and gender for the purpose of analyzing sample uniformity.

Statistical analyses were carried out using SPSS 21.0 software [52].

## 3. Results

### 3.1. Performance in the AVLT-LP Trials, Delayed Recall (A7) and Gain Score as a Function of Age Range, Gender and Cognitive Status

**Figure 1:**
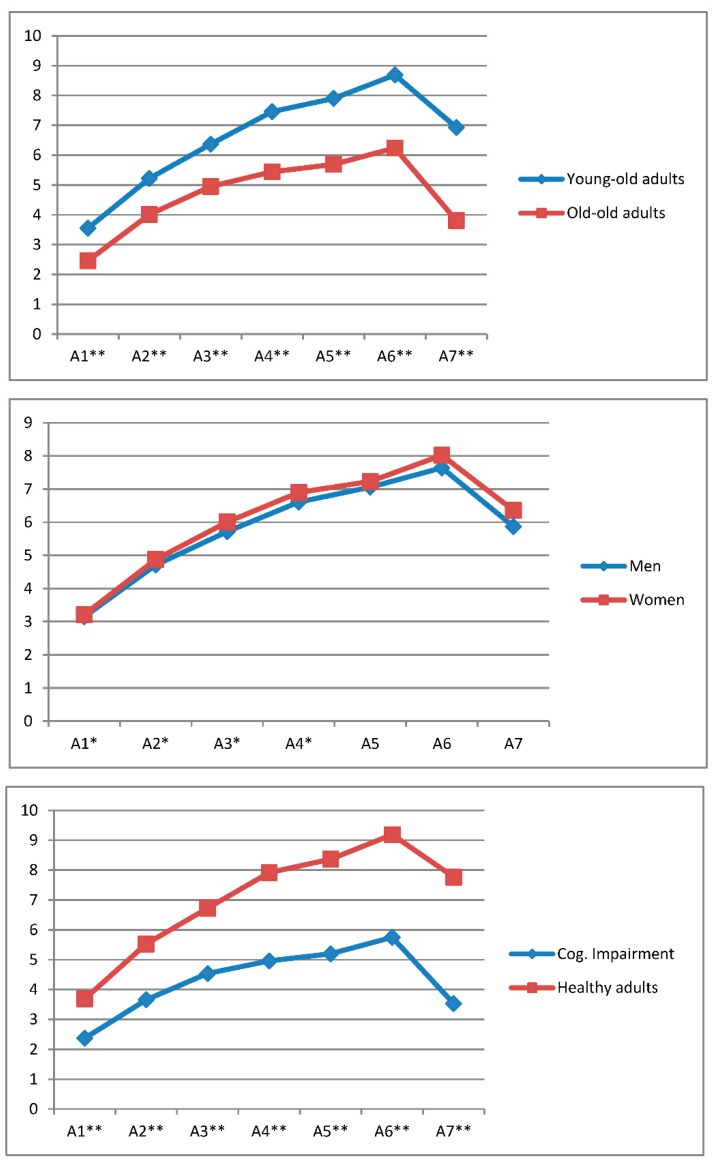
Participants’ learning curves and delayed recall (A7) as a function of age range, gender, and cognitive status. * The difference is significant at 0.05 (*p <* 0.05). ** The difference is significant at 0.01 (*p* < 0.01).

First, performance on the AVLT-LP test was analyzed for participants classified according to the following variables: Age range, gender, and cognitive status (See Table 1 and Figure 1).

Age range: The data showed significant differences between the two age groups on all trials of the AVLT-LP (A1: F_(1/568)_ = 62.98, *p* < 0.001, ŋ^2^ = 0.1; A2: F_(1/568)_ = 48.988, *p* < 0.001, ŋ^2^ = 0.08; A3: F_(1/568)_ = 45.578, *p* < 0.001, ŋ^2^ = 0.074; A4: F_(1/568)_ = 67.126, *p* < 0.001, ŋ^2^ = 0.106; A5: F_(1/568)_ = 66.811, *p* < 0.001, ŋ^2^ = 0.105; A6: F_(1/568)_ = 72.870, *p* < 0.001, ŋ^2^ = 0.114), in delayed recall (A7: F_(1/568)_ = 45.695, *p* < 0.001, ŋ^2^ = 0.117), and in the gain score (AVLT-LP: F_(1/568)_ = 35.801, *p* < 0.001, ŋ^2^ = 0.059), with the young-old group scoring higher in all cases. In all cases, the observed power was 1 and the effect size was medium (ŋ^2^ between 0.06 and 0.14) except for a low effect size in the AVLT-LP gain score (ŋ^2^ = 0.059).

Gender: The results show that while the women’s mean performance was somewhat higher than the men’s, the differences were significant only in A1 (F_(1/568)_ = 5.448, *p* < 0.05, ŋ^2^ = 0.000), A2 (F_(1/568)_ = 5.837, *p* < 0.05, ŋ^2^ = 0.002), A3 (F_(1/568)_ = 6.546, *p* < 0.05, ŋ^2^ = 0.003), and A4 (F_(1/568)_ = 3.882, *p* < 0.05, ŋ^2^ = 0.002). There were no significant differences between men and women in trials A5 (F_(1/568)_ = 2.612, *p* > 0.05, ŋ^2^ = 0.001) and A6 (F_(1/568)_ = 2.429, *p* > 0.05, ŋ^2^ = 0.003). Neither were there significant differences between men and women in delayed recall (A7) (F_(1/568)_ = 1.438, *p* > 0.05, ŋ^2^ = 0.004) or in gain score (F_(1/568)_ = 0.731, *p* > 0.05 ŋ^2^ = 0.001). The effect size was low in all cases (ŋ^2^ < 0.01) and the observed power was close to 1.

Cognitive status: Results showed significantly higher performance levels in persons without cognitive impairment in all test trials. The mean differences between the groups became increasingly larger with successive applications of the test trials (A1: F_(1/568)_ = 53.511, *p* < 0.001, ŋ^2^ = 0.155; A2: F_(1/568)_ = 72.748, *p* < 0.001, ŋ^2^ = 0.2; A3: F_(1/568)_ = 66.728, *p* < 0.001, ŋ^2^ = 0.188), especially between the second training trial (A4) (F_(1/568)_ = 90.434, *p* < 0.0001, ŋ^2^ = 0.240) and the post-training phase (A5 and A6) (A5: F_(1/568)_ = 88.355, *p* < 0.001, ŋ^2^ = 0.231; and A6: F_(1/568)_ = 5.448, *p* < 0.001, ŋ^2^ = 0.238). In delayed recall (A7), significant differences were also found in favor of the group of healthy adults (F_(1/568)_ = 66.110, *p* < 0.0001, ŋ^2^ = 0.274), with a significant drop in the score of the cognitive impairment group. For the gain score, significant between-group differences were also found in favor of healthy adults (F_(1/568)_ = 48.189, *p* < 0.001, ŋ^2^ = 0.131). The effect size was medium in A1 (ŋ^2^ = 0.056) and high in all the other test indices (ŋ^2^ > 0.14), with an observed power of 1 in all cases.

### 3.2. Distribution of the Participants According to Their Age Range, Cognitive Status, Gender and Cognitive Plasticity Status

Second, we analyzed the distribution of participants according to their plasticity status (calculated from the Schöttke et al. algorithm [51], their age range, gender and cognitive status. As shown in Table 2, the distribution was not homogeneous in the case of age range (χ^2^ = 26.027 _(2/268)_ *p* < 0.0001), such that the highest percentage of persons with low plasticity was found in the 81+ group (63.68%). In the case of gender, the sample showed homogeneous distribution (χ^2^ = 0.476 _(1/268)_ *p* > 0.05), with a similar percentage of persons having high and low plasticity in the groups of men and women. As for cognitive status, the distribution was not homogeneous (χ^2^ = 50.063 _(1/268)_ *p* < 0.0001), such that 62.78% of the total group of persons with high plasticity did not have cognitive impairment, while 67.74% of persons with low plasticity presented cognitive impairment. Likewise, in the group of persons without cognitive impairment, 75.95% were classified with high plasticity and 52.87% of the group with cognitive impairment were classified with low plasticity.

### 3.3. Performance in the AVLT-LP: Comparing the Young-Old Adults and the Old-Old Adults with and without Cognitive Impairment

The analyses carried out (see Table 3) showed that the multivariate contrasts were significant for age range (Wilks’ Lambda *Λ*_(1*/*4)_ = 8.587, *p* < 0.001) and for cognitive status (Wilks’ Lambda: *Λ*_(1*/*4)_ = 23.601; *p* = <0.0001). The interaction between both factors was not significant (Wilks’ Lambda: *Λ*_(1*/*4)_ = 2.016; *p* > 0.05).

When cognitive status was compared, the intrasubject effects were significant for all the AVLT-LP measures (A1: F_(1/4)_ = 60.66, *p* < 0.001, ŋ^2partial^ = 0.003; A2: F_(1/4)_ = 92.96, *p* < 0.001, ŋ^2partial^ = 0.001; A3: F_(1/4)_ = 81.05, *p* < 0.001, ŋ^partial2^ = 0.001; A4: F_(1/4)_ = 116.94, *p* < 0.001, ŋ^2partial^ = 0.000; A5: F_(1/4)_ = 112.78, *p* < 0.001, ŋ^2partial^ = 0.001; A6: F_(1/4)_ = 122.38, *p* < 0.001, ŋ^2partial^ = 0.004; A7: F_(1/4)_ = 7.59, *p* < 0.05, ŋ^partial2^ = 0.051; A7 − A1: F_(1/4)_ = 3.76, *p* < 0.05, ŋ^2partial^ = 0.026; AVLT-LP gain score: F_(1/4)_ = 59.378, *p* < 0.001, ŋ^2partial^ = 0.096). The effect size was low from A1 to A7 (ŋ^2^ < 0.01) and high for A7 − A1 and AVL-LP gain score (ŋ^2^ > 0.14). The Bonferroni correction showed all differences to be significant (*p* < 0.01).

When age range was compared, the intrasubject effects were significant for all the AVLT-LP measures except for the A7 − A1 index (A1: F_(1/4)_ = 33.42, *p* < 0.001, ŋ^2partial^ = 0.056; A2: F_(1/4)_ = 21.57, *p* < 0.001, ŋ^2partial^ = 0.037; A3: F_(1/4)_ = 18.84, *p* < 0.001, ŋ^partial2^ = 0.033; A4: F_(1/4)_ = 33.37, *p* < 0.001, ŋ^2partial^ = 0.056; A5: F_(1/4)_ = 33.42, *p* < 0.001, ŋ^2partial^ = 0.056; A6: F_(1/4)_ = 38.77, *p* < 0.001, ŋ^2partial^ = 0.065; A7: F_(1/4)_ = 4.515, *p* < 0.05, ŋ^partial2^ = 0.03; AVLT-LP gain score: F_(1/4)_ = 16.826, *p* < 0.001, ŋ^2partial^ = 0.148; A7 − A1: F_(1/4)_ = 0.616, *p* > 0.05, ŋ^2partial^ = 0.004). The effect size was low from A1 to A5 and for A7 and A7 − A1 (ŋ^2^ < 0.06), medium for A6 (ŋ^2partial^ = 0.065) and high for AVLT-LP gain score (ŋ^2partial^ = 0.148). The Bonferroni correction showed all differences to be significant (*p* < 0.001).

Although the interaction between the two factors (age range and cognitive status) was not significant in the MANOVA, one may cautiously assert that the ANOVA was significant for the following measures: A4 (F_(1/4)_ = 7.209, *p* < 0.05, ŋ^2partial^ = 0.013), A5 (F_(1/4)_ = 10.719, *p* < 0.001, ŋ^2partial^ = 0.019), A6 (F_(1/4)_ = 9.402, *p* < 0.05, ŋ^2partial^ = 0.017) and AVLT-LP gain score (F_(1/4)_ = 9.915, *p* < 0.05, ŋ^2partial^ = 0.017). In these cases, the effect size was low (ŋ^2^ < 0.06).

## 4. Discussion

The main objective of the present study was to analyze cognitive plasticity as assessed through the AVLT-LP, in a large sample of older people, and to analyze how that cognitive plasticity relates to variables reported in the specialized literature to have an influence, namely, age range (young-old adults and old-old adults), gender and cognitive status [29,30,45,53].

The results showed significant between-group differences in performance and in the learning curve (trials A1 to A6) in favor of the younger group, who significantly outperformed the over 80 group. These results agree with findings from other studies where learning potential continues to decline with age [4,6,37,54] and with studies that show good levels of cognitive performance in young-old adults [35,36]. Differences also appeared in delayed recall (A7) and in AVLT-LP gain score, our indicator of cognitive plasticity, but in this case it was the over 80 group that showed significantly lower performance than the young-old adults group. It seems in this case that age 80 marks the start of a significant decline in cognitive plasticity and in long-term memory, as suggested by prior research that places the transition from the third to the fourth age at about 80–85 years [28,31,32,37,38]. These data are corroborated by our sample distribution according to age group and cognitive plasticity status (calculated from the Schöttke et al. algorithm [51], where we found that the greatest percentage of persons with low plasticity was in the over 80 group (43.52%).This classification is consistent with reports from prior research indicating that, while plasticity continues to be present at advanced ages (in our study 36.31% of the over 80 group presented plasticity), it is present to a lesser degree than in earlier stages [28,29,30].

In relation to cognitive status, results showed significantly higher performance levels in the healthy elders. Moreover, these differences increased over the duration of the test, with a very significant drop in delayed recall in persons with cognitive impairment. These data are in line with prior studies that indicate important differences in cognitive plasticity between persons with and without cognitive impairment, thereby showing the effectiveness of dynamic assessment procedures for identifying persons with cognitive impairment [20,25,26,45]. Additionally, results for delayed recall confirm that it has an important role in identifying persons with risk of dementia [45]. These authors propose that having a delayed recall score (trial A7) that is lower than one’s score in the first trial (A1) would be a clear indicator of Alzheimer-type dementia [45].

The interaction between age range and cognitive status should also be highlighted: The difference between the four groups was significant in the variables that involved learning ability, that is, A4, A5, A6 and AVLT-LP gain score. It also showed that healthy old-old adults had higher scores in those variables than young-old adults with cognitive impairment. These results confirm previous research regarding the presence of cognitive plasticity in old-old adults [29,30]. Decrease in cognitive plasticity appeared to be associated mainly with cognitive impairment, such that the presence of cognitive impairment would be the determinant of less plasticity, and not age range. From our point of view, this result is very significant and should be taken into account in future studies in this topic area.

Regarding gender, results from this study showed that -after controlling for educational level—the women presented slightly better performance than the men in the first four test trials (pre-test and training phases), with no differences in the last two trials, in delayed recall or in gain score (indicative of plasticity). Likewise, looking at our sample distribution, we found a similar percentage of persons with high and low plasticity in the groups of men and of women. Prior studies had reported better performance levels in episodic memory tasks in women [25,55,56,57]; this might be related to greater verbal ability in women [58] or, as Speer et al. [59] indicate, with biological factors, such as greater vascular risk in men, or an earlier onset of atrophy in the left-medial temporal lobe. In our study, notwithstanding, the differences between men and women are very slight and are not present in delayed recall or in cognitive plasticity, suggesting that there are no gender differences in ability to learn or in long-term memory in old age, after controlling for educational level. This result would confirm previous studies, such as Faille [60], where no gender differences were found in cognitive plasticity in a sample of elders with a mean age of 80 years.

When we analyze the participants classified according to their plasticity and cognitive status, we find the expected higher proportion of high plasticity persons in the group of healthy elders (75.94%). However, in the group of persons with cognitive impairment, we also found that 47.12% benefitted from the training given in the AVLT-LP test, significantly improving their performance on the post-test, and thereby showing cognitive plasticity.

This result is consistent with prior studies [3,22,26,61] that indicate the presence of plasticity in elders with cognitive impairment and also indicate the possibilities for using this type of measure when planning cognitive interventions and predicting the cognitive evolution of elders [22,26,44,62].

In short, the study presented here contributes more evidence of the importance of evaluating cognitive plasticity in the elderly, and offers information about the influence of variables, such as age, gender, and cognitive status, in a widely used task for assessing verbal memory and plasticity [57,63,64]. We consider this fact to be quite relevant, since it underscores the importance of considering variables, such as those analyzed here when evaluating an older population. Variables like age range and gender influence cognitive performance; consequently, specific data should be established as a function of these variables. In addition, the large sample used in this investigation allows the conclusions to be generalized to broader population samples. However, there are certain limitations to the study, for example, that the participants did not have an external diagnosis to confirm the presence or absence of impairment or dementia, and that while the sample is quite large, place of residence was uniformly a retirement home. It would also have been interesting to assess the participants with a test of executive control, due to its connection with cognitive plasticity [27]. Given these limitations, future studies should seek to work with samples of elders that reside in their own homes, and to analyze the differences between elders living in community settings and in senior residences, and who have a clinical diagnosis. Nonetheless, we believe that the data presented here may be of interest to the scientific community, to the extent that it offers information about the utility of the AVLT-LP for assessing the ever-increasing proportion of elders in our society today.

## Figures and Tables

**Table 1:** Mean scores and differences as a function of the variables analyzed in all Auditory Verbal Learning Test—Learning Potential (AVLT-LP) test trials and in AVLT-LP gain score.

AVLT-LP TRIALS	A1	A2	A3	A4	A5	A6	A7	Gain Score
AGE RANGE								
60–80 (*n* = 379)	3.55 ± 1.64	5.22 ± 2.04	6.37 ± 2.55	7.46 ± 2.95	7.90 ± 3.25	8.69 ± 3.32	6.93 ± 3.82	3.89 ± 2.26
81+ (*n* = 223)	2.46 ± 1.35	4.01 ± 1.74	4.95 ± 1.92	5.44 ± 2.37	5.69 ± 2.53	6.24 ± 2.78	3.81 ± 3.35	2.74 ± 1.99
F_(1/568)_	62.980 **	48.988 **	45.578 **	67.126 **	66.811 **	72.870 **	45.695 **	35.801 **
ŋ^2^	0.100	0.080	0.074	0.106	0.105	0.114	0.117	0.059
observed power	1.00	1.00	1.00	1.00	1.00	1.00	1.00	1.00
GENDER								
Men (*n* = 219)	3.15 ± 1.63	4.71 ± 2.00	5.72 ± 2.30	6.61 ± 2.83	7.06 ± 3.07	7.64 ± 3.31	5.87 ± 3.63	3.42 ± 2.12
Women (*n* = 350)	3.21 ± 1.64	4.88 ± 2.05	6.01 ± 2.54	6.90 ± 2.98	7.23 ± 3.28	8.02 ± 3.48	6.36 ± 4.14	3.57 ± 2.31
F_(1/568)_	5.448 *	5.837 *	6.546 *	3.882 *	2.616	2.429	1.438	0.731
ŋ^2^	0.000	0.002	0.003	0.002	0.001	0.003	0.004	0.001
observed power	0.847	0.872	0.908	0.701	0.521	0.489	0.307	0.100
COGNITIVE STATUS								
Cognitive impairment (*n* = 217)	2.37 ± 1.42	3.66 ± 1.60	4.54 ± 1.80	4.96 ± 2.07	5.20 ± 2.36	5.75 ± 2.66	3.53 ± 2.71	2.47 ± 1.91
Healthy adults (*n* = 352)	3.69 ± 1.55	5.53 ± 1.94	6.73 ± 2.43	7.92 ± 2.81	8.37 ± 3.05	9.19 ± 3.17	7.77 ± 3.71	4.15 ± 2.19
F_(1/568)_	53.511 **	72.748 **	66.728 **	90.434 **	88.355 **	93.312 **	66.101 **	48.189 **
ŋ^2^	0.155	0.200	0.188	0.240	0.231	0.238	0.274	0.131
observed power	1.00	1.00	1.00	1.00	1.00	1.00	1.00	1.00

* The difference is significant at 0.05 (*p <* 0.05). ** The difference is significant at 0.01 (*p* < 0.01).

**Table 2:** Contingency table according to plasticity status and the variables of age range, gender and cognitive status.

Plasticity Status	Age range		Gender		Cognitive Status	
	60–80	81+	Men	Women	Cognitive Impairment	Healthy Adults
Low plasticity	157	121	111	167	147	70
High plasticity	222	69	108	183	131	221
	χ^2^ = 26.027 *p* < 0.0001		χ^2^ = 0.476 *p* > 0.05		χ^2^ = 50.063 *p* < 0.0001	

**Table 3:** Multivariate Analysis of Variance (MANOVA, 2X2 Age range X cognitive status).

		Old-Old Adults		Young-Old Adults		Cognitive Status			Age Range			Interaction		
AVLT-LP TRIALS	Group	*M*	*SD*	*M*	*SD*	F_(1,4)_	η^2 *partial*^	O.P.	F_(1,4)_	η^2 *partial*^	O.P.	F_(1,4)_	η^2 *partial*^	O.P.
A1	Cog. Im	2.04	1.31	2.65	1.44	60.66 **	0.003	0.164	33.42 **	0.056	1.00	0.1776	0.003	0.265
	Healthy	2.97	1.23	3.93	1.58									
A2	Cog. Im	3.36	1.58	3.90	1.58	92.96 **	0.001	0.102	21.57 **	0.037	0.996	1.873	0.003	0.277
	Healthy	4.79	1.63	5.78	1.97									
A3	Cog. Im	4.29	1.60	4.75	1.95	81.05 **	0.001	0.127	18.84 **	0.033	0.991	0.3317	0.008	0.555
	Healthy	5.77	2.43	7.06	2.48									
A4	Cog. Im	4.59	1.82	5.29	2.24	116.94 **	0.000	0.082	33.37 **	0.056	1.00	7.209 *	0.013	0.764
	Healthy	6.48	2.56	8.48	2.72									
A5	Cog. Im	4.84	2.12	5.46	2.46	112.78 **	0.001	0.142	33.42 **	0.056	1.00	10.719 **	0.019	0.905
	Healthy	6.68	2.75	8.93	2.99									
A6	Cog. Im	5.27	2.43	6.10	2.75	122.38 **	0.004	0.318	38.77 **	0.065	1.00	9.402 *	0.017	0.865
	Healty	7.36	2.74	9.80	3.97									
A7	Cog. Im	3.08	2.08	4.09	2.61	7.59 *	0.051	0.782	4.515 *	0.03	0.560	0.350	0.002	0.090
	Healthy	4.90	2.85	6.51	3.44									
A7 − A1	Cog. Im	1.35	2.33	1.43	2.38	3.76 *	0.026	0.487	0.616	0.004	0.122	0.507	0.004	0.109
	Healthy	2.30	2.45	3.07	2.79									
AVLT-LP gain score	Cog. Im	2.36	1.79	2.53	1.94	59.37 **	0.096	1.000	16.826 **	0.148	0.984	9.915 *	0.017	0.882
	Healty	3.14	2.08	4.49	2.13									

* The difference is significant at 0.05 (*p <* 0.05). ** The difference is significant at 0.01 (*p* < 0.01). O.P. observed power.
